# Clipperton Atoll as a model to study small marine populations: Endemism and the genomic consequences of small population size

**DOI:** 10.1371/journal.pone.0198901

**Published:** 2018-06-27

**Authors:** Nicole L. Crane, Juliette Tariel, Jennifer E. Caselle, Alan M. Friedlander, D. Ross Robertson, Giacomo Bernardi

**Affiliations:** 1 Department of Biology, Cabrillo College, Aptos, CA, United States of America; 2 Department of Ecology and Evolutionary Biology, University of California Santa Cruz, Santa Cruz, California, United States of America; 3 Marine Science Institute, University of California Santa Barbara, Santa Barbara, CA, United States of America; 4 Pristine Seas, National Geographic Society, Washington, DC, United States of America; 5 Fisheries Ecology Research Lab, Department of Biology, University of Hawaii, Honolulu, HI, United States of America; 6 Smithsonian Tropical Research Institute, Balboa, Panamá; Department of Agriculture and Water Resources, AUSTRALIA

## Abstract

Estimating population sizes and genetic diversity are key factors to understand and predict population dynamics. Marine species have been a difficult challenge in that respect, due to the difficulty in assessing population sizes and the open nature of such populations. Small, isolated islands with endemic species offer an opportunity to groundtruth population size estimates with empirical data and investigate the genetic consequences of such small populations. Here we focus on two endemic species of reef fish, the Clipperton damselfish, *Stegastes baldwini*, and the Clipperton angelfish, *Holacanthus limbaughi*, on Clipperton Atoll, tropical eastern Pacific. Visual surveys, performed over almost two decades and four expeditions, and genetic surveys based on genomic RAD sequences, allowed us to estimate kinship and genetic diversity, as well as to compare population size estimates based on visual surveys with effective population sizes based on genetics. We found that genetic and visual estimates of population numbers were remarkably similar. *S*. *baldwini* and *H*. *limbaughi* had population sizes of approximately 800,000 and 60,000, respectively. Relatively small population sizes resulted in low genetic diversity and the presence of apparent kinship. This study emphasizes the importance of small isolated islands as models to study population dynamics of marine organisms.

## Introduction

Populations of marine organisms typically are very large, with population sizes (N) of 10^6^ to 10^9^ individuals being common [1]. Large populations in an open environment are predicted to show high levels of gene flow, resulting in low genetic population structure and speciation rates. An early paradox arose from the observation of elevated speciation rates and strong population structure in marine organisms, despite few physical barriers (i.e. preventing allopatric speciation), and large populations being the norm (i.e. countering neutral drift) [1].

The discovery of limited dispersal in marine systems, based on otolith microchemistry and genetic parentage analyses, offered a potential answer to this paradox, showing that populations, once thought to be open, often experience high levels of self-recruitment [2–6]. It is therefore likely that many populations that are ecologically or numerically large, are effectively much smaller in genetic terms due to inbreeding as a long-term consequence of self-recruitment. In general, effective population size (Ne), the average number of individuals per generation contributing genes to the next generation is smaller, with Ne/N typically ranging between 0.1 and 0.5, but sometimes reaching much smaller values of 10^−5^ and even lower [7–10]. However, most such populations (with very small Ne/N ratios) are difficult to study because estimating population sizes of marine organisms can be a challenge [11]. Endemic populations on small and isolated atolls offer an opportunity to estimate population sizes more readily, and to investigate the prevalence of low genetic diversity and inbreeding depression [12–18]. The general aim of this research was to study fish species with presumed small population sizes (N) and estimate their effective population sizes (Ne), levels of inbreeding, and relative genetic diversity, in order to assess the evolutionary consequences of small population sizes. We used such an approach on Clipperton Atoll, investigating two of its endemic reef fishes: the Clipperton angelfish, *Holacanthus limbaughi* Baldwin 1963, and the Clipperton damselfish, *Stegastes baldwini* Allen and Woods, 1980. Due to the habitat limitations in this spatially restricted system, comparing visual survey estimates and genetic estimates of population sizes was an attainable goal.

### Clipperton Atoll

**Fig 1:**
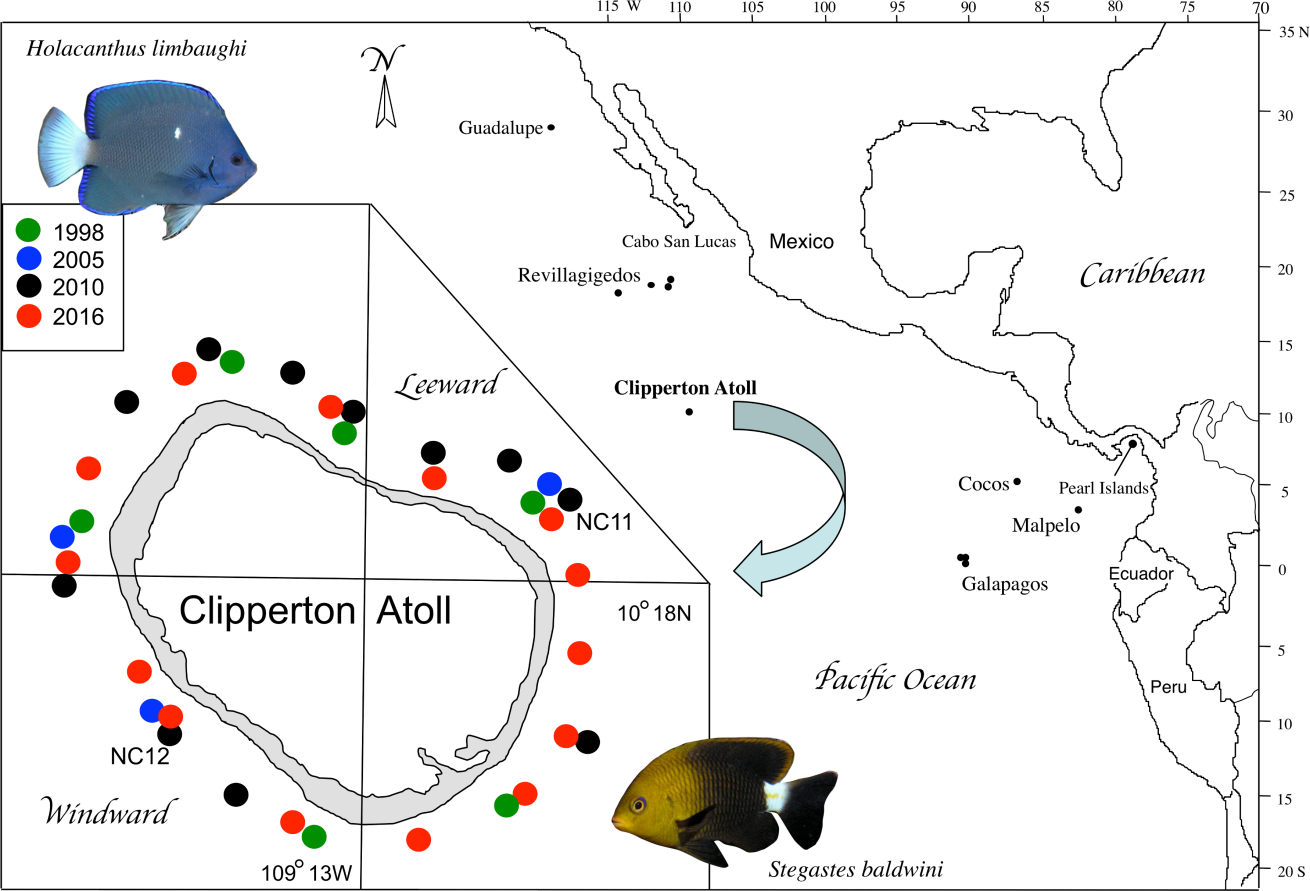
Map of Clipperton Atoll and adjacent areas. Inset shows a detailed map of the Atoll and the sampling sites of the four different expeditions. Fish and benthos were surveyed at all sites in 1998, 2005 and 2016. In 2010, benthos was surveyed at all sites, and fish were surveyed at the labeled sites NC11 and NC12.

Clipperton Atoll is an isolated atoll in the tropical eastern Pacific (TEP) that is approximately 1300 km to the west of mainland Mexico, and 1000 km south of the Revillagigedo Archipelago (Fig 1). Clipperton Atoll is relatively small, with an emergent land surface of approximately 6 km^2^, and a circumference of approximately 12 km [19,20]. It is surrounded by an almost continuous coral-rich fringing reef (approximately 50% live coral cover), with high fish biomass (356.6 g m^–2^) [21]. Clipperton is the only atoll in the TEP and the only site within that region in which all habitat for reef fishes is provided by corals and their soft-bottom derivatives. There are 156 reef-associated fish species recorded from Clipperton, 104 of them with resident populations [22], and seven of those being endemic, a proportion similar to that found on other TEP oceanic islands [22–25]. The seven endemic reef fish species include, *Myripristis gildi*, *Ophioblennius clippertonensis*, *Pseudogramma axelrodi*, *Thalassoma robertsoni*, *Xyrichtys wellingtoni* and the two focal species of this study, *H*. *limbaughi* and *S*. *baldwini*. Studies made on small-island systems (including Clipperton Atoll) have proposed common characteristics of endemic fishes including small body sizes and limited dispersal potential, effectively “trapping” endemics due to the distance between oceanic islands and other distant reefs [26–28].

### *Holacanthus limbaughi* and *Stegastes baldwini*

The Clipperton angelfish, *H*. *limbaughi*, belongs to a genus that is found in the eastern tropical Atlantic (1 species), western tropical Atlantic (3 species), and the TEP (3 species) [29,30]. The TEP species form a monophyletic group that includes three closely related species: the king angelfish, *H*. *passer*, whose distribution is the widest, from Mexico to Ecuador, including the Galàpagos and Cocos islands; and two insular species, the Clipperton angelfish, *H*. *limbaughi*, which is restricted to Clipperton Atoll [31], and the Clarion angelfish, *H*. *clarionensis*, which is mostly found at the Revillagigedo Islands and sporadically at the southern tip of the Baja California Peninsula. A single vagrant adult of this species was sighted at Clipperton in 1994, but not again in subsequent expeditions [23,25].

The Clipperton damselfish, *S*. *baldwini*, belongs to a widespread damselfish genus of approximately 40 species that occurs in the Atlantic and the Indo-Pacific, with eight species in the TEP. *S*. *baldwini* belongs to a complex of three primarily insular, closely related species that include the whitetail damselfish, *S*. *leucorus*, which is primarily found at the Revillagigedo Islands, Rocas Alijos, and Guadalupe Island, and is occasionally found on the mainland from southern California to Mazatlan, Mexico, and the Galàpagos ringtail damselfish, *S*. *beebei*, which is primarily found in the Galàpagos Islands, but is also found commonly on the islands of Malpelo and Cocos, and as a vagrant in continental Costa Rica, Panamà, Ecuador and Peru [18,23,31]. *S*. *baldwini* is the only member of its genus that has ever been recorded at Clipperton, and it has not been recorded at any other locations.

The two focal species of this study exhibit some life history and ecological differences, in addition to having very different sizes. *H*. *limbaughi* has a maximum length of 25 cm, while *S*. *baldwini* is much smaller with a maximum length of 9 cm. *H*. *limbaughi* primarily feeds on benthic, sessile invertebrates, including sponges, while the smaller *S*. *baldwini* is primarily omnivorous, feeding on algae and small invertebrates.

Spawning has not been recorded for *H*. *limbaughi*, however, it was observed for the closely related *H*. *passer* which spawns in pairs or small harems [32]. Males aggregate in lek-like formations, and the mating results in broadcasting tens of thousands of pelagic eggs daily, with pelagic larval duration likely being around 25 days [32]. In contrast, like other neotropical members of the genus, *S*. *baldwini* probably spawns in pairs on the benthos, and males guard a nest with hundreds of eggs until they hatch and pelagic larvae are released [33,34]. The pelagic larval duration is on average 26 days [35]. Both sequential hermaphroditism and skewed sex ratio have been shown to affect the variance of reproductive success, which in turn greatly affects effective population size [36]. While some angelfishes in other genera are protogynous hermaphrodites with female biased sex ratios [37], a study on *H*. *passer*, a close relative of *H*. *limbaughi*, could not find definitive evidence of such hermaphroditism. However, that study did find that smaller individuals were predominantly females and the largest fish exclusively males, and that the overall sex ratio was slightly female biased (1.32:1) [38]. In contrast there is no evidence that any species of *Stegastes* is hermaphroditic, it is therefore assumed that this is also the case for *S*. *baldwini*.

The goal of this study was to capitalize on a system where endemic species of diurnally active reef fishes are found on a very small, highly isolated reef, thus providing a model of manageable size to assess population sizes using visual censuses and genetic tools. We used visual surveys on scuba to estimate fish population size, and Restriction site Associated DNA (RAD) genomic sequencing to assess population size based on genetic approaches. We then tested for inbreeding and lowered genetic diversity to investigate the genomic consequences of small population sizes.

## Materials and methods

### Extent of habitat and population size estimates

For depths within diving range (0-20m), benthic surveys were performed at 11 sites around the island in 2010 (black circles, Fig 1) to assess habitat availability, using point intercept approaches. Below those depths, a remotely operated vehicle (ROV) (in 1998), and a submersible (2016) were used to assess the presence or absence of the focal species. The extent of planar reef habitat was then plotted on a bathymetric map of Clipperton Atoll. A copy of that bathymetric map is provided in S1 Fig. This was derived from chart 21641 of the Defense Mapping Agency that was supplemented by soundings made by the Smithsonian Tropical Research Institute’s Research Vessel, RV Urraca, during the 1998 expedition.

All fish were counted and were identified to the species level. The transect count area was along a 50 meter long transect on a 5 m wide swath, and extended from the sea floor to the surface of the water column following established protocols [39,40]. Scuba transect counts of the numbers of individuals of each species were done on three separate expeditions, in 1998, 2010 and 2016, by subsets of the authors, and these data were supplemented with published data collected in 2005 [21] (Fig 1). Counts were analyzed separately, and coded as shallow (between 6m and 12m depth), and deep (20m depth) strata.

Transects described in the methods yielded estimates of fish densities. To obtain an estimate of population size based on fish densities, we first calculated the population size estimated for each sampling year by multiplying the density values with the available reef habitat. We then followed Vucetich et al.’s [40] recommendation of using the harmonic mean over several sampling years to estimate the population size of each species, thus taking into account natural population fluctuations [41].

### Ethics statement

All samples were collected under the permit 460 given by the Delegation Régionale à la Recherche et à la Téchnologie (DRRT) of the Haut-Commisariat de la République en Polynésie Francaise. Collections followed University of California Santa Cruz Institutional Animal Care and Use Committee (IACUC) protocol Berng1701.

### Sample collections

Samples of *H*. *limbaughi* (N = 35) and *S*. *baldwini* (N = 24) were collected at Clipperton Atoll using pole spears while scuba diving. In order to assess the taxonomic positions of our focal species, we used sister species *H*. *clarionensis* (1 sample—Aquarium of the Pacific, 4 samples—San Benedicto, Revillagigedo Archipelago), *H*. *passer* (2 samples—Galàpagos Archipelago, 12 samples—Panama, and 16 samples—Mexico), *S*. *leucorus* (3 samples—Guadalupe Island, 8 samples—San Benedicto, Revillagigedo Archipelago), and *S*. *beebei* (5 samples—Galàpagos Archipelago, 1 sample Cocos Island). A description of these samples can be found in two previously published papers [18,29]. Fin clip tissue samples were preserved in 95% ethanol immediately after collection and stored at room temperature until reaching the lab where they were placed in a -20^0^ C freezer.

### DNA extractions and RAD libraries

DNA was extracted using DNeasy Blood & Tissue kits (Qiagen) according to manufacturer’s protocol. We constructed RAD libraries using a variation of the original protocol with restriction enzyme SbfI [42–45]. Individually barcoded samples were sequenced on an Illumina HiSeq 2500 at the Vincent J. Coates Genomics Sequencing Laboratory at the University of California Berkeley.

Raw 100 bp reads were trimmed to 92 bp on the 3’ end, quality filtered, and then split according to the 6 bp unique barcode using custom Perl scripts [42] (also available in S1 Script and S2 Script). Sequences were dropped if the product of quality scores for their 92 bases was below 80%. The barcode (6 bp) and restriction site residue (6 bp) were then removed from the 5’ end, resulting in a final sequence length of 80 bp.

We used the software program Stacks version 1.29 [46,47] to identify orthologous sequences. We first ran the program *denovo_map*.*pl*, which runs all three Stacks components in a pipeline (i.e., ustacks, cstacks, and sstacks). Parameter optimizations followed general published guidelines [48]. We set a minimum stack depth (-m) of three, a maximum of three mismatches per loci for each individual (-M), and allowed up to seven mismatches when building catalog loci (-n). Minor allele frequencies were kept at the default value of 0.05 [48]. We then ran the Stacks program *populations* to generate output files for input into downstream phylogenetic programs, retaining all SNPs. Due to high coverage across individuals, we increased the minimum stack depth (-m) to eight in *populations* runs. We created a stringent dataset by setting -p at 7, which means all individuals must retain the marker and with -r set to 80%, meaning that 80% of the individuals must retain the marker. All Fastq sequence files are available from the GenBank at the National Center for Biotechnology Information short-read archive database (accession number: SRP136950). Associated metadata are also available at GeOMe (GUID https://n2t.net/ark:/21547/BAK2, https://n2t.net/ark:/21547/BAY2) [49].

### Genetic analyses

We used outputs of the population script of Stacks to create different types of infiles. Genetic diversity of each group was estimated using Arlequin [50]. There is a slight bias on genetic diversity when using RAD sequences due to the biased selection of sequences near common restriction sites [51]. In addition, this bias is further reduced when dealing with populations with low genetic diversity, which is the case here with small populations restricted to a small island [51].

In order to assess introgression between sister species, we used a Bayesian approach implemented in Structure 2.2 [52,53] to analyze genetic clusters using structure files created by the population script of Stacks. We used a burn-in of 10 000, under the admixture model, with prior population information included to assist clustering.

Considering that population sizes are presumably small for our focal species, there is a potential for related individuals to be present in our dataset. To test for potential relatedness, we calculated kinship coefficients [54] for each pair of individuals using Genodive [55]. These coefficients are based on the probability of identity of two alleles for each pair of homologous genes compared between each pair of individuals. Kinship was estimated with respect to the allele frequencies for the full data set, so these coefficients provide an index of relative relatedness between each pair of individuals based on default settings in Genodive. To estimate different degrees of relatedness, we used the Loiselle et al.’s [52] co-ancestry coefficients (full- sib = 0.25, half-sib = 0.125) to generate the following bins: ‘nearly identical’ (0.57 > k > 0.375), ‘full-sib’ (0.374 > k > 0.1875), ‘half-sib’ (0.1874 > k > 0.09375) and ‘quarter-sib’ (0.09374 > k > 0.047).

With the advent of full genome sequencing, Ne estimates have capitalized on physical genomic maps to best assess the variation of effective population size through time by estimating variations of precise linkage disequilibrium based on the physical map [56–59]. The lack of a physical map for our non-model species precludes such approaches at this time. Here, we estimated Ne using two approaches. First, we estimated Ne using a linkage disequilibrium method in the absence of a map [60] with bias correction [61], and incorporation of missing data [62] as implemented in NeEstimator V2.01 [63]. Values of linkage disequilibrium (r^2^) for each locus pair were first generated with NeEstimator with a minor allele frequency cutoff of 0.05. It is considered that locus pairs with r^2^ > 0.5 are likely to be physically linked, which may bias estimates downward [64]. We found, however, that only two pairwise comparisons had such r^2^ values; therefore no corrections were necessary in our dataset [65]. Second, we obtained Ne by estimating the value π (Pi) using Stacks. Values of π are correlated with both Ne and mutation rates (when in neutral equilibrium π = 4Neμ [66,67]. The mutation rate (μ) is expressed as mutation rate per site per generation. Mutation rate for RAD sequences in fish has been estimated at 10^−8^ to 10^−9^ mutations per site [68,69]. Generation time was not available for our focal species, we therefore used data for the closest available relatives [70]: 4.1 years for *Pomacanthus semicirculatus* (*Pomacanthus* is an angelfish genus closely related to *Holacanthus*, [71]), and 2.6 years for *Stegastes acapulcoensis*, a TEP congener to *S*. *baldwini* [72].

## Results

### Population size estimates based on visual census

Several sites were surveyed to assess the suitability of habitat around the atoll as shown in Fig 1. At a subset of sites, fishes were counted on transects that were performed both on the windward and leeward sides of the atoll during three separate expeditions in 1998, 2010, and 2016, at 6, 2, and 14 sites, respectively (Fig 1). In addition, fish abundance data were supplemented by data obtained from the 2005 expedition (blue dots, Fig 1) [21]. A total of 73 independent transects were performed over all years, 47 of them in shallow strata (between 6m and 12m depth), and 26 in deeper strata (20m depth).

Estimates of density from visual surveys for *H*. *limbaughi* and *S*. *baldwini* varied significantly with year and depth but not site (described in detail below). However, in all cases data consistently showed that the density of the two species differed by approximately one order of magnitude (Table 1). The average density over four sampling years for *H*. *limbaughi* was 164 ind.ha^-1^ (individuals per hectare), while the average density for *S*. *baldwini* was 3,064 ind.ha^-1^ (Table 1).

**Table 1:** Estimates of abundance, genetic diversity, kinship, and population size for *Holacanthus limbaughi* and *Stegates baldwini* at Clipperton Atoll.

**Abundance**			Visual Counts		
	1998	2005	2010	2016	Average
*H*. *limbaughi*	61	168	190	237	164
*S*. *baldwini*	806	1856	2425	7171	3064.5
**Genetic diversity**	n	Stacks	Stacks	Arlequin	Arlequin
		Polymorphic	π (Pi)	obs. het	exp. het.
		loci			
*H*. *limbaughi*	35	6.66%	0.0009	0.04	0.05
*S*. *baldwini*	24	3.55%	0.0008	0.11	0.18
**Kinship**			Genodive		
	Identical	Full-sibs	Half_sibs	Quarter sibs	
*H*. *limbaughi*	35	5–0.79%	55–8,73%	88–13.97%	
(630 pairwise comparisons)					
*S*. *baldwini*	24	1–0.33%	7–2.33%	46–15.33%	
(300 pairwise comparisons)					
**Population size**	Visual counts		Tajima π (Pi)		NeEstimator (LD)
	estimated N		Ne		Ne
*H*. *limbaughi*	3.57 10^4^–6.44 10^4^		5.48 10^3^–54.8 10^3^		109.0 –inf.
*S*. *baldwini*	48.7 10^4^–56.5 10^4^		7.69 10^4^–76.9 10^4^		375.9 –inf.

Abundance estimates from visual counts are in number of individuals per hectare. Number of positive sibship comparisons are provided with their percentage over the total number of comparisons. For example 5 full-sibship comparisons were found in *H*. *limbaughi*, which correspond to 0.79% of all 630 pairwise comparisons.

**Fig 2:**
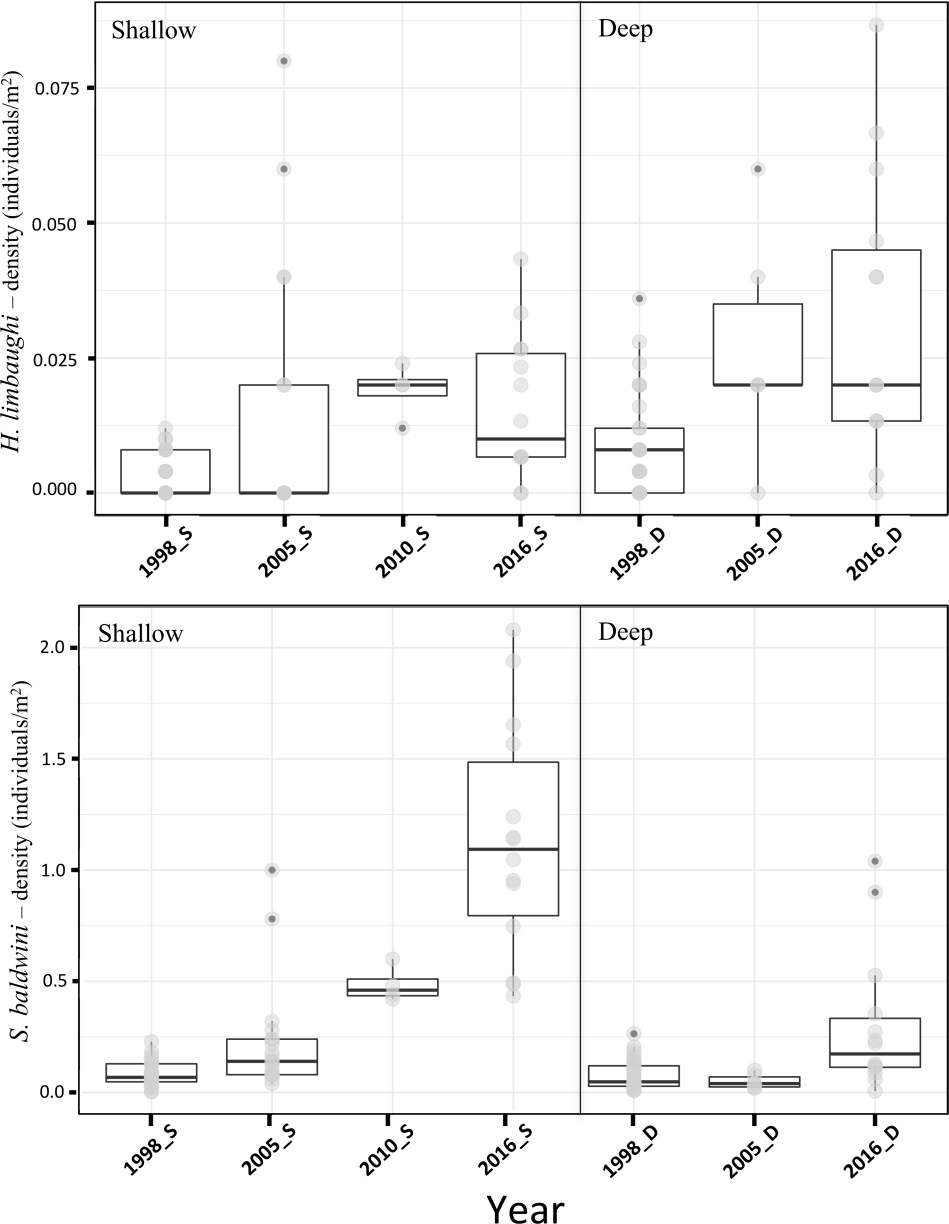
Fish densities. Density (number per m^2^) from visual census for *Holacanthus limbaughi* and *Stegastes baldwini* shown as box plots separated by both year of census and depth of census.

There was significant inter-annual variation in density for both species (one way ANOVA, *H*. *limbaughi* p = 0.026; *S*. *baldwini* p<0.0001) (S2 Fig). Both species showed a trend towards increased density from 1998 to 2016. Density estimates for transects performed at different depths (shallow and deep) were also found to be statistically different, with greater density in shallow water (t-test, *H*. *limbaughi* p = 0.009, *S*. *baldwini* p = 0.004) (S3 Fig). Finally, when considering both depth and year of sampling as factors, density estimates were not found to be statistically different for *H*. *limbaughi* (one way ANOVA, shallow transects p = 0.141; deep transects p = 0.084). In contrast, for *S*. *baldwini*, deep and shallow transects were found to be statistically different (one way ANOVA, shallow transects p = 0.049, deep transects p<0.0001) (Fig 2). In addition, for *S*. *baldwini*, an increase in density over the years in shallow water showed a slightly better match for an exponential increase over a linear increase (Akaike Information Criterion AICc = 45.21 and AICc = 47.53 for exponential and linear increase, respectively) (Fig 2).

Deep surveys using ROV dives (in 1998) and manned submersible dives (in 2016) showed that large populations of *H*. *limbaughi* are found consistently to a depth of approximately 100 m, while *S*. *baldwini*, which occurs to 100 m, showed low densities below 60 m. We considered all coral reef substrate as suitable habitat for the species studied here, as there is only one sand patch of appreciable size (~20m diameter) on the reef above ~50m depth. Benthic visual assessments showed coral reef habitat to at least 50 m depth. Based on our hydrographic map, reef habitat to a depth of 50m was estimated to cover approximately 2.838 km^2^ (see S1 Fig). Deeper habitat between 50-100m, comprised an additional 2.287 km^2^ (see S1 Fig).

Considering only shallow water habitat (to 50m) and the calculated population sizes for each sampling year, the harmonic mean of these values resulted in an estimated total population size for *H*. *limbaughi* of approximately 35,600 at 0–50 m depth, increasing to 64,400 when considering the additional habitat down to 100 m. The latter value is consistent with the estimate of 61,000 presented in the literature [28]. Population size for shallow *S*. *baldwini* was approximately 490,000 individuals. We estimated a reduction of density of *S*. *baldwini* by a factor of 5 in deeper water (50–100 m). Hence, adding habitat to 100 m depth for that species only increased the estimated census number to 565,000 individuals (Table 1).

### RAD sequencing

RAD sequencing produced similar sequence yields for the two studied species. For *Holacanthus*, our approach yielded an average 49,454 loci per individual, with 4210 polymorphic loci and 5557 SNPs. For *Stegastes*, our approach yielded an average 47,898 loci per individual, with 3016 polymorphic loci and 4582 SNPs.

### Inter-specific gene flow and introgression

**Fig 3:**
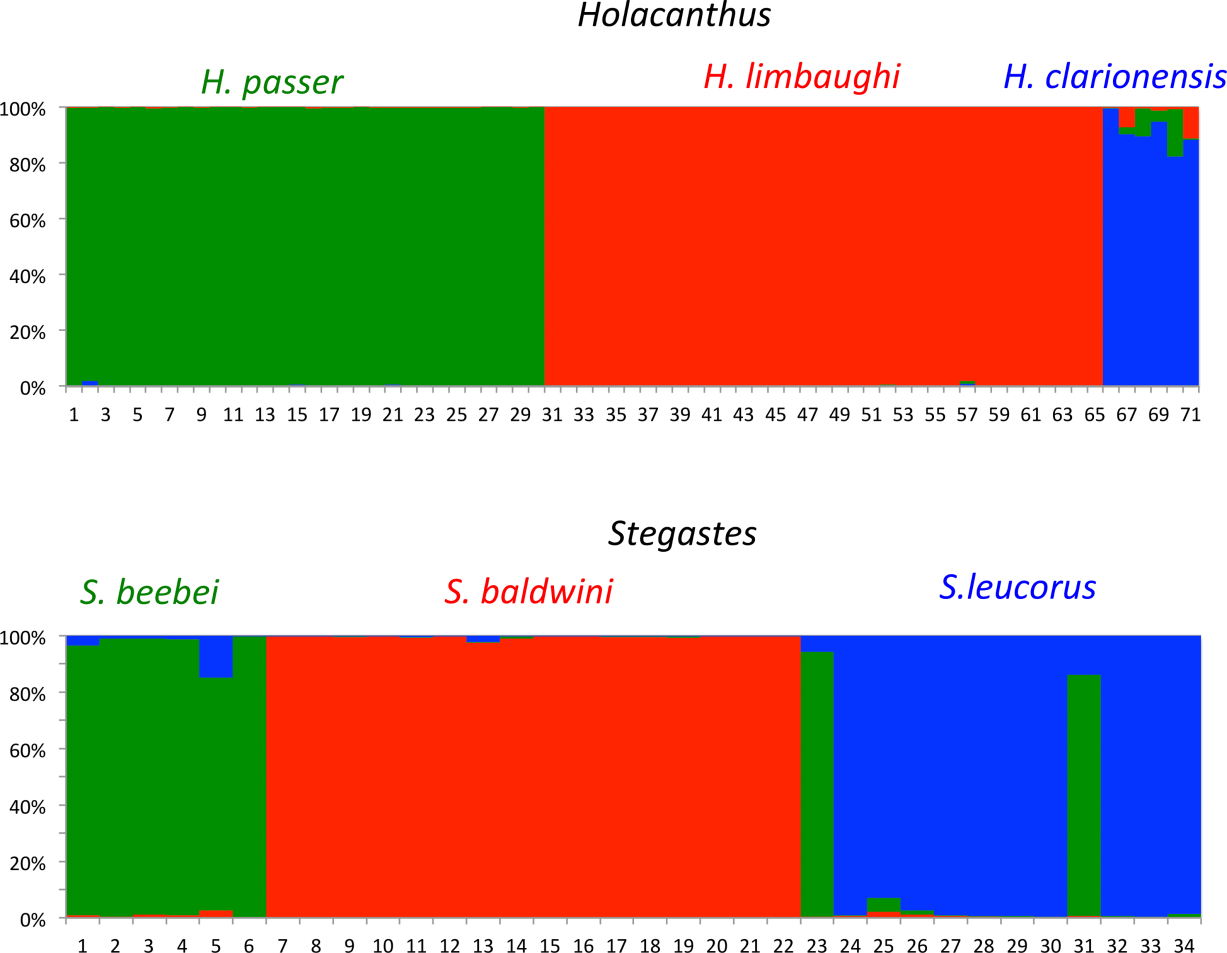
Structure plots of *Holacanthus* and *Stegastes*. Structure plots of closely related species *Holacanthus passer*, *H*. *clarionensis*, and *H*. *limbaughi* (top panel); and *Stegastes beebei*, *S*. *leucorus*, and *S*. *baldwini* (bottom panel); based on RAD seq molecular markers.

At the outset, we wanted to make sure that the species used in this study were not being introgressed by sister species that could potentially bias our estimates of population size, inbreeding, and genetic diversity. We therefore included samples from closely related species that might potentially contribute to the genome of our study species. A population analysis showed that there was no evidence of gene flow between our species and their respective sister species, as shown by a Structure analysis (Red clusters, Fig 3). There was some evidence of gene flow among non-focal sister species (for example between *Stegastes beebei* and *S*. *leucorus*, Fig 3) but these findings are not germane to this study and will be presented elsewhere.

### Genetic diversity

Estimates of genetic diversity are presented in Table 1. Since genetic diversity theoretically is positively correlated with population size [73,74], we expected *S*. *baldwini* to show higher levels of genetic diversity than *H*. *limbaughi*. This was indeed the case, both observed and expected heterozygosity were found to be 2.75 and 3.6 times greater, respectively, in *S*. *baldwini* compared to *H*. *limbaughi* (Table 1). When looking at polymorphic loci in stacks (which is different to heterozygosity, as it is a simple tally of polymorphic loci), we found that more loci were polymorphic in *H*. *limbaughi* compared to *S*. *baldwini* (6.66% of the loci, compared to 3.55%, respectively), suggesting that the distribution of polymorphisms not only differs in the two species, but does so in a reverse manner to heterozygosity.

### Kinship analyses

Kinship analyses based on RAD sequences showed high levels of kinship in *H*. *limbaughi* and *S*. *baldwini*. In these two species, we found 5 pairs (out of 35 individuals) and 1 pair (out of 24 individuals) of genetic full sibs, respectively (Table 1). Out of 630 pairwise comparisons, we found that 55 involved genetic half-sibs in *H*. *limbaughi*, while out of 300 comparisons, 7 involved genetic half-sibs in *S*. *baldwini*. All *H*. *limbaughi* individuals were involved in at least one genetic half-sib relationship, while 14 of 24 *S*. *baldwini* individuals were connected with other individuals through a half sibship relationship. When considering quarter sibships, all individuals for both species were connected with at least one other individual.

### Population size estimates based on genetic data

Effective population size estimates were determined using values of π (Pi) and direct values of Ne based on the LD method. Both approaches show population sizes to be greater in *S*. *baldwini* compared to *H*. *limbaughi*. Effective population size was found to be 14 times greater for S. baldwini than for H. limbaughi based on estimates of π (and corrected by the difference in generation times). When using estimates of Ne implemented by NeEstimator, populations of *S*. *baldwini* were found to be 3.4 times greater than for *H*. *limbaughi* (Table 1).

These analyses were repeated after removing pairwise comparisons involving full-sibling relationships (to avoid major genetic bias i.e. four comparisons for *H*. *limbaughi*, one for *S*. *baldwini*). The biological effect of this correction, however, was trivial. Corrected values of genetic diversity were also calculated and similarly remained virtually unchanged.

Effective population size estimates based on NeEstimator (LD method) resulted in very small population sizes, 109.0 and 375.9 individuals for *H*. *limbaughi* and *S*. *baldwini*, respectively (Table 1). However, for both species, the high end of the 95% confidence interval reached infinity, indicating that substantially larger effective population sizes cannot be excluded.

## Discussion

In this study, we capitalized on the presence of endemic reef fishes at a small, extremely isolated atoll to address the evolutionary consequences of small population sizes in marine organisms. In this study, we confirmed that there is no evidence of gene flow between *H*. *limbaughi* and its sister species, nor between *S*. *baldwini* and its sister species, and that the system is effectively closed from a genetic standpoint, thus validating endemism in these two species.

The two species used in this study are the only species in their respective genera at Clipperton Atoll, are easily identifiable because both have distinct coloration, do not behave cryptically, and do not resemble any other local species. Consequently, fish counts are likely to be accurate. Visual census data and genetic estimates of population size were consistent for both focal species. Census counts estimated that population size ratio between species are approximately one order of magnitude (*S*. *baldwini / H*. *limbaughi* = 18.6), a value very similar to what was obtained using genetic estimates based on Tajima’s π (ratio = 14). These ratios were consistent despite slight inter-annual variations in visual census values.

In general for marine populations, the ratio between N and Ne reflects differences of several orders of magnitude [8,75]. In contrast, we found that estimates of N from visual census versus estimates of Ne by Tajima’s Pi equation resulted in a ratio (Ne/N) between 0.85 and 0.14, was very similar to general stable populations where the ratio is approximately 0.14 [9,76], and thus was considerably higher than expected for marine populations. Indeed, marine fish populations have very low Ne/N ratios, such as the dark-blotched rockfish (Ne/N = 0.001–0.0001), the red drum (Ne/N = 0.001), the New Zealand snapper (1.8–2.8 10^−5^), and the Atlantic cod (3.9 10^5^) [77–80]. The use of the LD approach implemented in NeEstimator placed this ratio at 0.002 to infinity (Table 1), likely reflecting a bias in the method due to the lack of a physical genomic map. With a full and annotated genome, it is likely that this approach would provide a more accurate estimate for Ne.

In theory, effective population size is smaller than census population size, in some cases much smaller [8], a pattern that has been observed numerous times in empirical studies [81]. Similarly, in this study, values of N, based on visual censuses were greater than values of Ne based on genetic data. In addition, the censused population may have been underestimated due to a poor characterization of the available habitat, or due to some under-sampled habitat. This latter situation is likely because densities of individuals below 20 m that were seen in 1998 using ROV dives, and in 2016, while doing submersible dives, were not estimated. Indeed, we observed *H*. *limbaughi* at 110 m depth, and *S*. *baldwini* at 67 m and 72 m, in areas that were not surveyed using scuba (S4 Fig). We have tried to provide a best estimate of habitat availability based on our bathymetric knowledge of Clipperton, but this is admittedly a rather coarse estimate (S1 Fig). On the other hand, RAD sequencing approaches may bias the estimate of Ne, which, together with the absence of a physical map of the loci used in this study, increases the error in estimating Ne based on the LD method. Considering all these potential biases, it is remarkable to find such consistent population size estimates between visual and genetic approaches. Overall, our results confirm the greater abundance of *S*. *baldwini* compared to *H*. *limbaughi*, and provide consistent estimates for the numerical population sizes of these two species.

Small population sizes are theoretically associated with reduced genetic diversity due to random drift [13,74,82]. This was shown, for example, in *Stegastes sanctipauli*, a damselfish endemic to St. Peter and Paul’s Rocks, a tiny set of rocks nearly 1,000 km off northeast Brazil [83]. In our study, those expectations were met, where population size and genetic diversity were found to be very low for the endemics *H*. *limbaughi* and *S*. *baldwini* on Clipperton Atoll, which is about two orders of magnitude larger than St. Peter and Paul Rocks.

Indeed, both heterozygosity and amount of polymorphic loci were very reduced in these two species compared to species with much larger geographic distributions and population sizes. Heterozygosity values were extremely low for both species (0.05 and 0.18 for *H*. *limbaughi and S*. *baldwini*, respectively) with numbers comparable to the lowest recorded heterozygosities in naturally bottlenecked populations of cheetah (*Acinonyx jubatus*) and platypus (*Ornithorhynchus anatinus*) [13,84,85]. These heterozygosity values were also lower or similar to numbers obtained for local populations of other coral reef fishes, such as Red Sea and Oman anemonefish, genus *Amphiprion* (0.214–0.262), and Caribbean hamlets, genus *Hypoplectrus* (0.118–0.125) [86,87]. The number of polymorphic loci was also very small (6.7% and 3.6% for *H*. *limbaughi and S*. *baldwini*, respectively), with numbers again comparable with the number of polymorphic loci in the cheetah genome (approximately 5%) [84]. These numbers are also lower than observed for other RAD sequence data observed in typical coral reef fishes that have much larger geographic ranges and much larger population sizes (average 13.7%, range 10.2–19.2%, for *H*. *passer*, *Fistularia commersonii*, *Pomacentrus pavo*, *Stegastes nigricans*, *Pomachromis fuscidorsalis*, *Dascyllus aruanus*, *Chrysiptera brownriggii*, *Chromis margaritifer*, *Plectroglyphidodon lacrymatus*, *Chromis iomelas*, Bernardi, unpublished), and also lower than values for closely related congeneric species that have large ranges and population sizes, *H*. *passer* (10.2%) and *Stegastes nigricans* (12.7%) (Bernardi, unpublished).

The very low levels of genetic diversity described above unfortunately come with a practical consequence of lowering the power to detect true relatedness, because the lowered heterozygosity increases the sharing of alleles by chance alone. In this study, we found that a large proportion of individuals are apparently related (for example, we collected 5 and 1 full “siblings” for *H*. *limbaughi* and *S*. *baldwini*, respectively). Although these numbers seem surprising, considering that samples were collected randomly, they do make sense when one considers only genetic relatedness, rather than assigning classical values of kinship. Individuals are indeed genetically similar, but this may be due to the low levels of genetic diversity rather than true kinship, as has been observed in other studies of organisms in which inbreeding is rampant [88]. Such an interpretation is consistent with the fact that when the apparent full sibs are removed from the analyses, numbers for theta and genetic diversity do not change (see the Results section above). In a situation where sampling is biased and accidentally includes related individuals, removing those individuals should raise genetic values significantly due to the removal of sampling biases [89]; yet we have not observed this trend here. Our interpretation is that we did not remove *actual* siblings, but rather that all individuals share a large number of alleles and therefore represent *apparent* genetic siblings.

## Conclusion

The goal of this study was to empirically validate estimated Ne using genetic techniques, with visual census data for population size in small marine populations, and to investigate the consequences of small populations and closed systems on genetic diversity. We sought to capitalize on a unique system in the marine environment where endemic populations of reef fishes are exclusively found on a small remote island. The isolated Clipperton Atoll, at which we studied two of its seven endemic reef-fishes, *H*. *limbaughi* and *S*. *baldwini*, offered such an opportunity. Due in part to the limited spatial extent of habitat, and thus increased accuracy of diver surveys, the census data presented here were consistent across depths and years.

We found that neither species showed evidence of being introgressed by sister species present in other parts of the TEP, thus confirming their endemic status. Using visual census and RAD sequencing methods, we were able to compare results for estimated population sizes of those two species. Population size estimates based on visual surveys and genetic approaches were remarkably consistent. While some variability in diver counts was present, the ratios of N to Ne were consistent across sampling units. This is noteworthy as population sizes in marine species have historically been difficult to estimate, and even more difficult to validate. Genetic techniques have been widely used to estimate population size, but this is one of a few studies that was able to validate a genetic approach with visual census approach. Both species demonstrated small population sizes for marine organism and associated low genetic diversity. Low genetic diversity was evidenced by extremely low heterozygosity values, and high levels of apparent relatedness in both species. This has evolutionary consequences for species with similar life history and habitat constraints, and these results have practical applications for marine species and ecosystem management, particularly for relatively ‘closed’ systems.

These two species of fishes represent a unique model to study a fully closed system in a marine environment. The presence of additional endemic species at Clipperton also provides a roadmap to continue this investigation and identify more general trends. Small isolated islands have contributed significantly to our understanding of general evolutionary and ecological patterns [90,91]. Similarly, this study emphasizes the importance of these systems as models to study population dynamics of marine organisms, and to expand understanding through more thorough ecological investigations to illuminate drivers of life history patterns in these unique marine ecosystems.

## Supporting information

S1 Fig Bathymetric map of Clipperton Atoll that shows habitat estimated contours. Pink outline represents the 50 m “shallow” water, blue outline respesents the 100m “deep” water. Numbers on the map represent depths values in fathoms.
(PDF)

S2 Fig Density (number per m^2^) from visual census for *Holacanthus limbaughi* and *Stegastes baldwini* by year. Data shown as box plots by year of census.
(PDF)

S3 Fig Density (number per m^2^) from visual census for *Holacanthus limbaughi* and *Stegastes baldwini* by depth. Data shown as box plots by depth, shallow (6–12 m) and deep (20m).
(PDF)

S4 Fig Picture of a group of *Holacanthus limbaughi* taken from a submersible dive at 110 m depth in 2016 (Alan Friedlander). (JPG)

S1 Script Quality filtering perl script. (TXT)

S2 Script Barcode split perl script. (TXT)
