# Supplementary figures and images for: Clipperton Atoll as a model to study small marine populations: Endemism and the genomic consequences of small population size
Source: PLoS One. 2018 Jun 27;13(6):e0198901. doi: 10.1371/journal.pone.0198901 (PMC6021044; doi:10.1371/journal.pone.0198901)

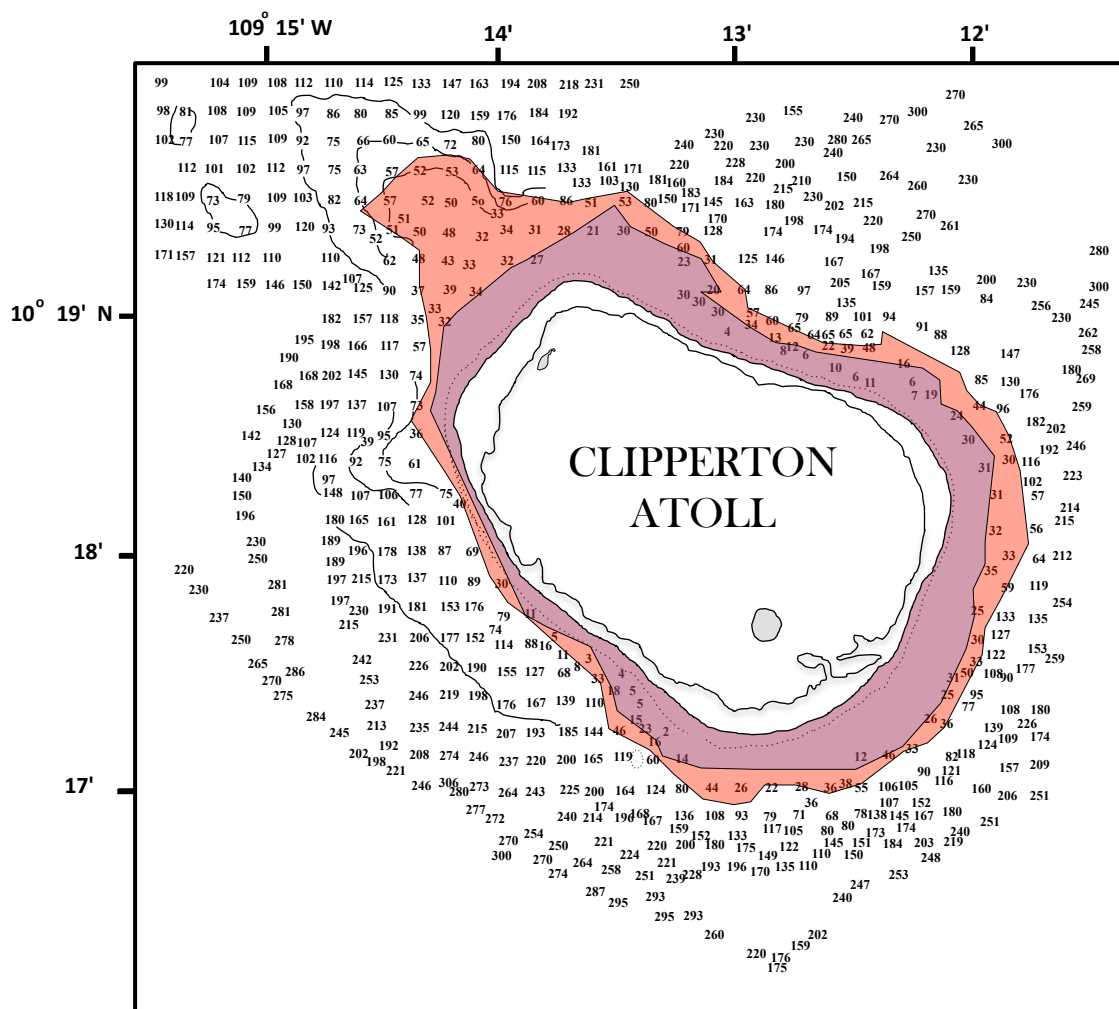

Supplementary Figure 1

Supplement: S1 Fig — Pink outline represents the 50 m “shallow” water, blue outline respesents the 100m “deep” water. Numbers on the map represent depths values in fathoms. (PDF) [file pone.0198901.s001.pdf]

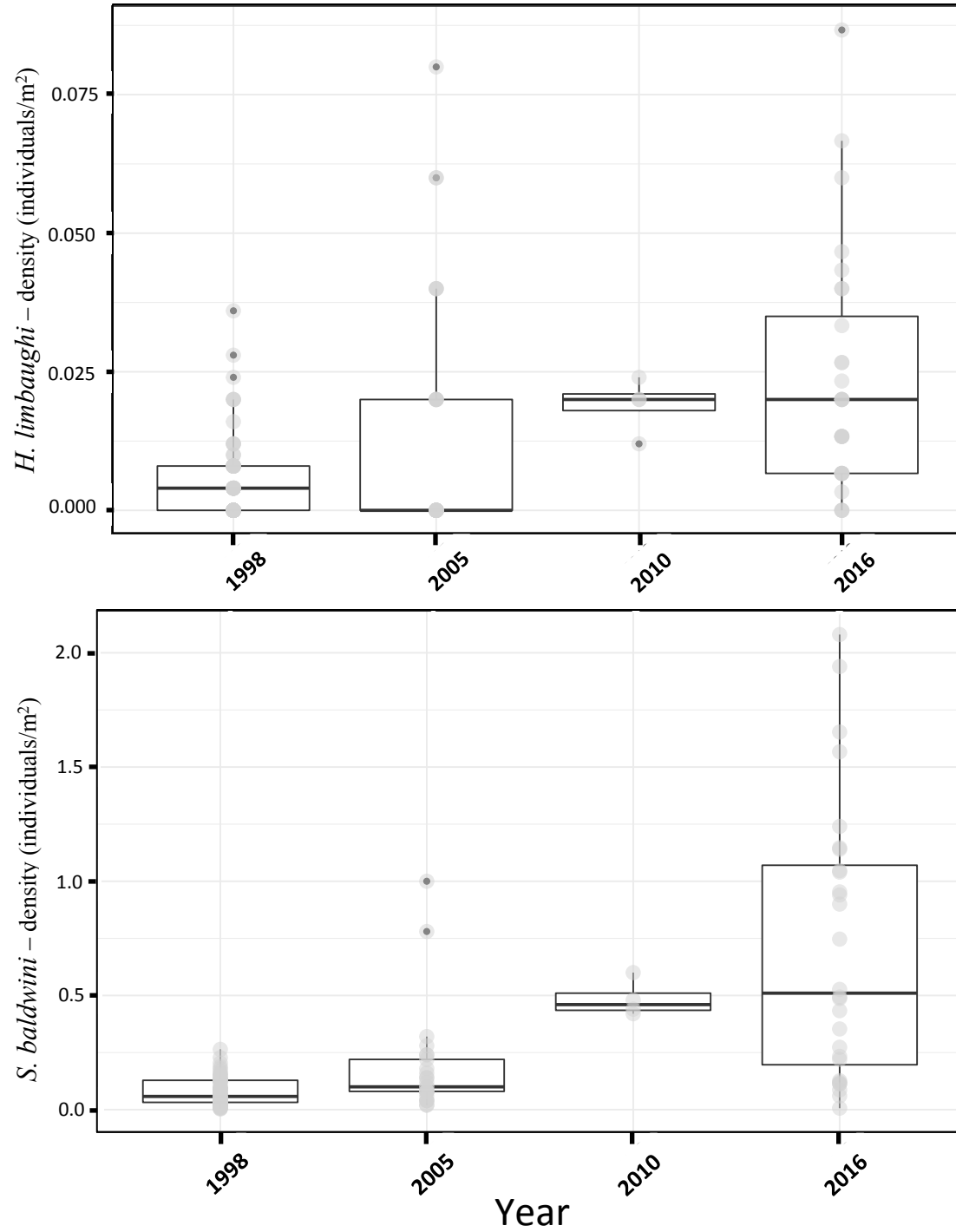

Supplementary Figure 2

Supplement: S2 Fig — Data shown as box plots by year of census. (PDF) [file pone.0198901.s002.pdf]

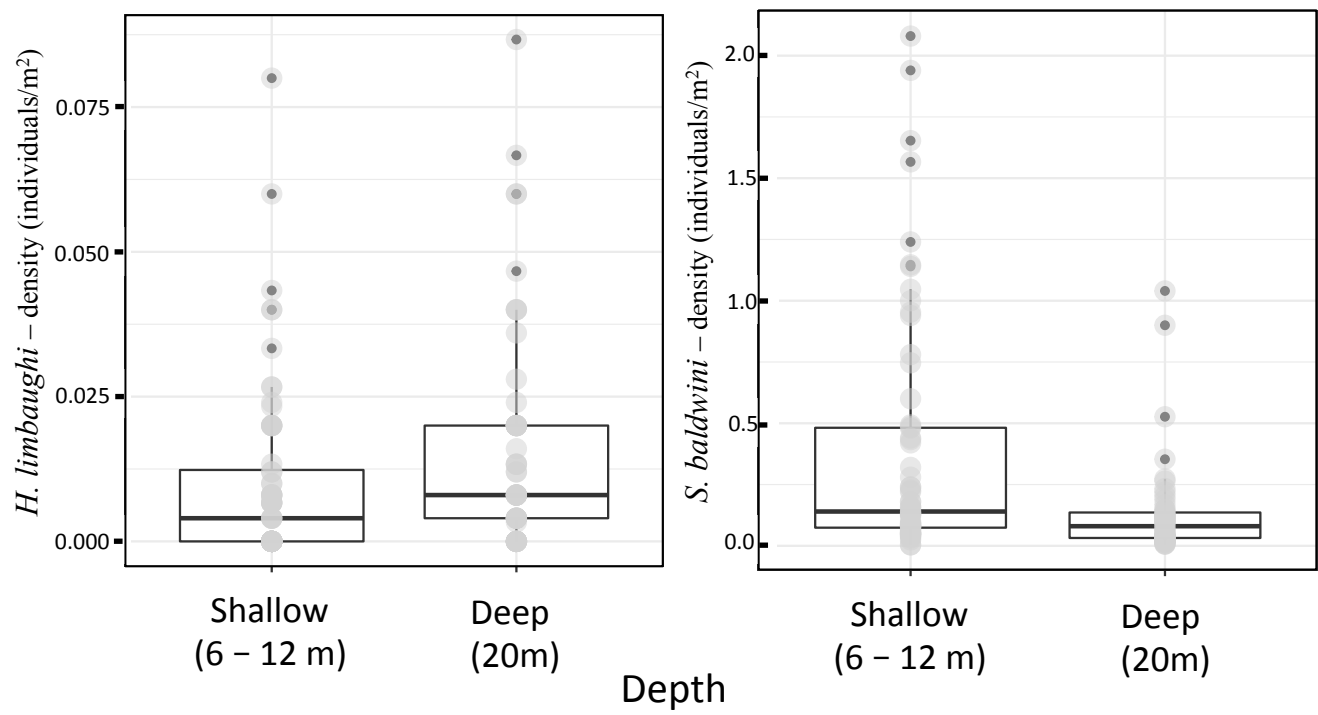

Supplementary Figure 3

Supplement: S3 Fig — Data shown as box plots by depth, shallow (6–12 m) and deep (20m). (PDF) [file pone.0198901.s003.pdf]

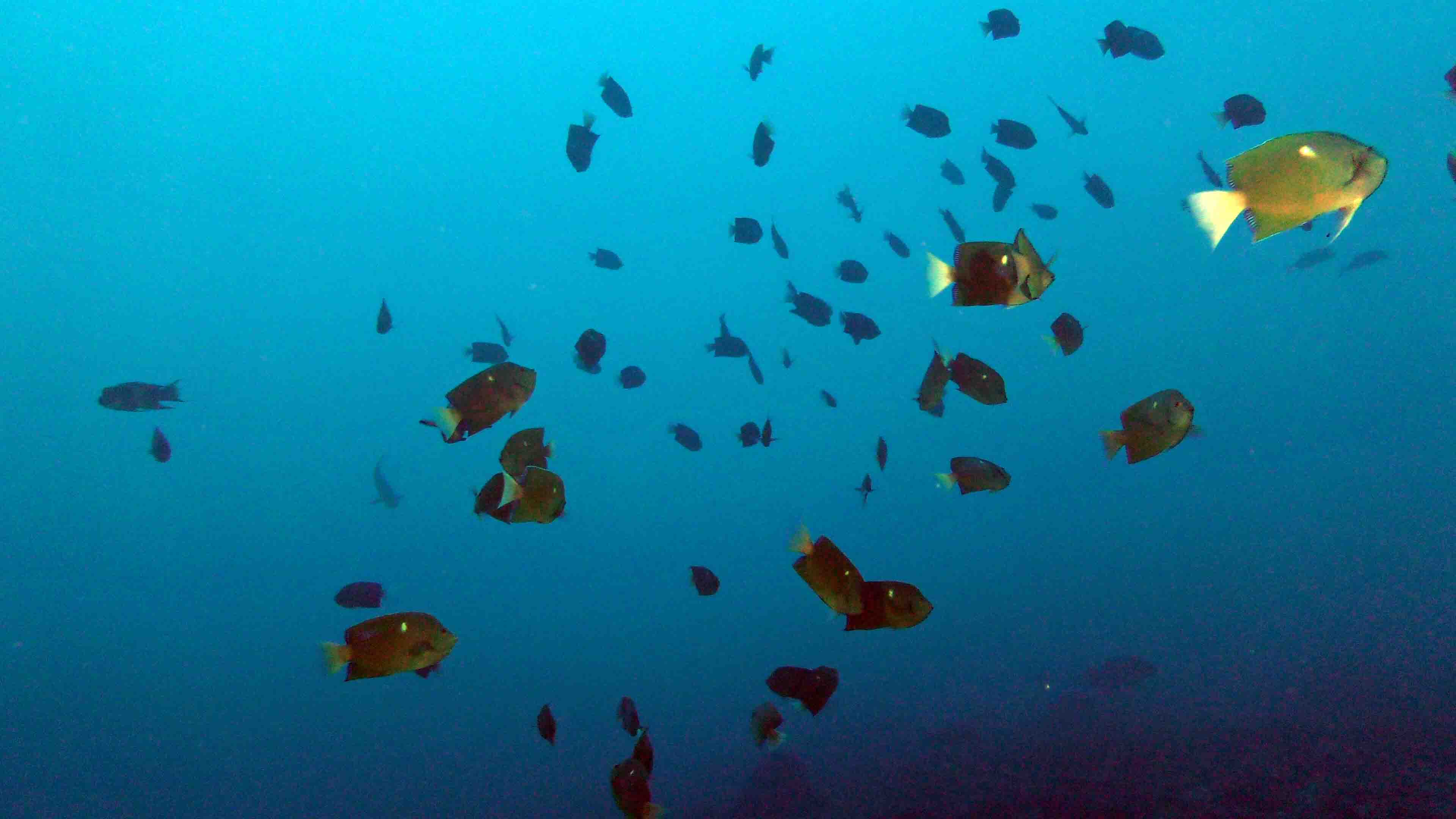

Supplement: S4 Fig — (JPG) [file pone.0198901.s004.jpg]
